# Identification of novel biomarkers and candidate small molecule drugs in rheumatoid arthritis and osteoarthritis based on bioinformatics analysis of high‐throughput data

**DOI:** 10.1042/BSR20193823

**Published:** 2020-12-16

**Authors:** Bin Zuo, JunFeng Zhu, Fei Xiao, ChengLong Wang, Yun Shen, XiaoDong Chen

**Affiliations:** Department of Orthopedic Surgery, Xinhua Hospital, Affiliated to Shanghai JiaoTong University School of Medicine (SJTUSM), Shanghai, China

**Keywords:** candidate small molecules, differentially expressed genes, osteoarthritis, rheumatoid arthritis

## Abstract

Background: Rheumatoid arthritis (RA) and osteoarthritis (OA) are two major types of joint diseases. The present study aimed to identify hub genes involved in the pathogenesis and further explore the potential treatment targets of RA and OA.

Methods: The gene expression profile of GSE12021 was downloaded from Gene Expression Omnibus (GEO). Total 31 samples (12 RA, 10 OA and 9 NC samples) were used. The differentially expressed genes (DEGs) in RA versus NC, OA versus NC and RA versus OA groups were screened using limma package. We also verified the DEGs in GSE55235 and GSE100786. Functional annotation and protein–protein interaction (PPI) network construction of OA‐ and RA‐specific DEGs were performed. Finally, the candidate small molecules as potential drugs to treat RA and OA were predicted in CMap database.

Results: 165 up-regulated and 163 down-regulated DEGs between RA and NC samples, 73 up-regulated and 293 down-regulated DEGs between OA and NC samples, 92 up-regulated and 98 down-regulated DEGs between RA and OA samples were identified. Immune response and TNF signaling pathway were significantly enriched pathways for RA‐ and OA‐specific DEGs, respectively. The hub genes were mainly associated with ‘Primary immunodeficiency’ (RA vs. NC group), ‘Ribosome’ (OA vs. NC group), and ‘Chemokine signaling pathway’ (RA vs. OA group). Arecoline and Cefamandole were the most promising small molecule to reverse the RA and OA gene expression.

Conclusion: Our findings suggest new insights into the underlying pathogenesis of RA and OA, which may improve the diagnosis and treatment of these intractable chronic diseases.

## Introduction

Osteoarthritis (OA) is characterized by degradation of articular cartilage and subchondral bone resulting in the rigidity deformity and dysfunction of the joints [1]. Rheumatoid arthritis (RA) is a complex, multi-systemic autoimmune disease that mainly has an effect on the flexible joints as well. Although the symptoms of RA are similar to OA, the pathological components for the synovial in RA is quite different with OA [2]. RA manifests as synovial cell hypertrophy and hyperplasia infiltrated with lymphocytes and inflammatory cells, whereas OA has less infiltration of leukocytic [3,4]. As two major types of joint diseases, RA and OA have high morbidity and disability rate especially among the elderly people [5]. In addition, due to the lack of effective treatment, RA and OA are clinically incurable and therefore create huge economic burden for both patients and society [6].

Most recently, many researches have conducted high-throughput methods to screen the genetic factors involved in RA and OA [7–10]. Therefore, several key genes and novel diagnostic markers have been identified for these diseases. However, prediction of small molecules targeting the gene expression of RA and OA and the underlying mechanisms of the two diseases remain far from being elucidated.

The present study aimed to identify hub genes involved in the pathogenesis of OA and RA and further explore the potential drug targets. First, we screened and verified the differentially expressed genes (DEGs) associated with RA and OA respectively from the downloaded gene expression profiles GSE12021, GSE55235 and GSE100786. Functional annotation and protein–protein interaction (PPI) network construction of RA‐ and OA‐specific DEGs were performed to further explore the molecular mechanisms underlying RA and OA. Most importantly, the Connectivity Map (CMap) database was used to explore candidate small molecule drugs potentially targeting RA and OA.

## Methods

### Data resources

**Figure 1:**
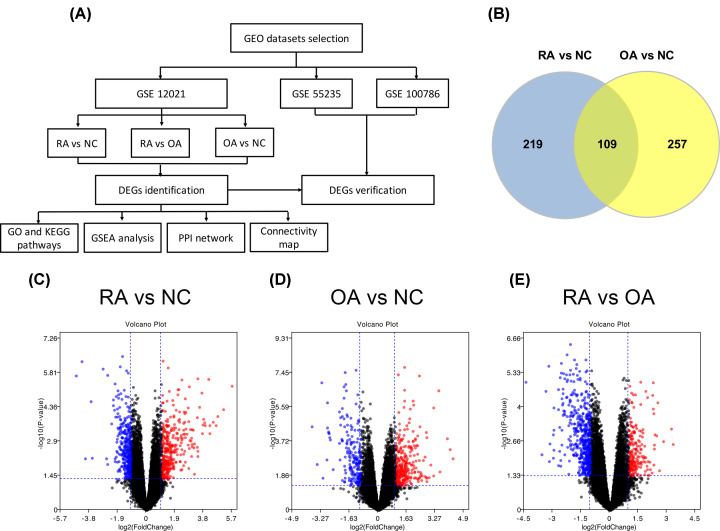
Identification of DEGs (**A**) Flow chart of the present study: data collection, processing, analysis, and validation. (**B**) Venn diagram of DEGs in RA and OA. Blue represented DEGs between RA and NC and yellow represented DEGs between OA and NC. Volcano plot of gene expression profile data between RA and NC (**C**), OA and NC (**D**), RA and OA (**E**). Red dots: significantly up-regulated genes; Blue dots: significantly down-regulated genes; Black dots: nondifferentially expressed genes. *P*<0.05 and |log2 Foldchange |>1 were considered as significant; DEG, differentially expressed gene; OA, osteoarthritis; RA, rheumatoid arthritis; NC, normal controls.

Gene expression profile dataset GSE12021, GSE55235 and GSE100786 was downloaded from the GEO database (http://www.ncbi.nlm.nih.gov/geo/) (Figure 1A). The dataset GSE12021, GSE55235 and GSE100786 were produced by Affymetrix Human Genome U133A Array (Agilent Technologies, Santa Clara, CA). The dataset GSE12021 contained data of synovial tissues from 31 samples, including 9 normal control (NC), 12 RA, and 10 OA samples. The dataset GSE55235 is including data of synovial tissues from 10 RA and 10 OA samples. The dataset GSE100786 included 6 human peripheral blood (PB) and 8 bone marrow (BM) monocytes samples from patients with RA and OA, respectively.

### Data reprocessing

We downloaded the raw data files from GEO datasets. R language (version 3.4.3) (http://www.r-project.org/) and Bioconductor (http://bioconductor.org/) were used to perform data analysis. Data normalization was performed using the Affy package in R software and every probe set was normalized.

### Differential expressed genes identification

The normalized data were intersected with gene symbol and reference information. And then the data were analyzed with the Limma package in the R in order to examine DEGs. Only the genes with *P* value ≤ 0.05 and |log2 fold change (FC)| ≥ 1 were identified as DEGs. Differential expressed genes with statistical significance were identified through volcano plot filtering. Hierarchical clustering was performed using Morpheus (https://software.broadinstitute.org/morpheus/).

### GO and pathway enrichment analysis of DEGs

GO and KEGG pathway enrichment analyses were performed to explore the biological characteristics and functional annotation of candidate DEG using the online tool DAVID (https://david.ncifcrf.gov/). The genes in modules were also analyzed in the same way. Additionally, the Networks Gene Oncology tool (BiNGO) plugin in Cytoscape was used to perform and visualize the biological process analysis of the DEGs.

### Protein–protein interaction network construction and hub gene identification

PPI network was constructed by the Search Tool for the Retrieval of Interacting Genes database (STRING, https://string-db.org/). The Molecular Complex Detection (MCODE) in Cytoscape software was performed to screen the functional modules in the PPI network. Moreover, hub genes were determined based on the interaction edges in the network. Also, GO and KEGG enrichment analyses were performed in module genes as mentioned earlier.

### Gene set enrichment analysis

Gene set enrichment analysis (GSEA) could detect changes in the gene set rather than the individual genes, so it can uncover these subtle expression changes and is expected to yield more desirable results. In order to study the effect of the DEGs on various biological functions, GSEA (v3.0, http://software.broadinstitute.org/gsea/downloads.jsp) was used to analysis DEGs of biological functional annotation and pathways.

### Identification of small molecules

We queried the Connectivity Map (CMap, http://www.broadinstitute.org/cmap/) to screen the candidate small molecule drugs based on the gene signature of RA and OA. CMap is a collection of databases that stores thousands of gene transcription-expression profiles from cultured mammalian cells exposed to active small molecule drugs. The DEGs were divided into up-regulated and down-regulated groups. The enrichment scores ranging from −1 to +1 were calculated, which represented the similarity. A positive connectivity value (closer to +1) revealed that a small molecule is able to induce the gene expression of RA and OA, whereas a negative connectivity value (closer to −1) reveled that a small molecule is able to imitate the status of normal cells.

## Results

### Identification and verification of DEGs

The DEGs were investigated in RA, OA and NC in GSE12021.A total of 328 genes were identified to be differentially expressed between RA and NC samples with the threshold of *P*<0.05 and a minimal 1-fold change of expression. Among these DEGs, 165 were up-regulated and 163 down-regulated in RA compared with NC samples. The top 10 up- and down-regulated genes for RA and NC are listed in Table 1. Similarly, 366 DEGs were identified to be differentially expressed between OA and NC samples, including 73 up-regulated and 293 down-regulated genes. In addition, a total of 190 DEGs between RA and OA were identified, including 92 up-regulated and 98 down-regulated genes. The top 10 DEGs for OA versus NC and RA versus OA samples are listed in Tables 2 and 3, respectively. The Venn diagrams showed the 109 overlap DEGs between DEGs of RA versus NC and DEGs of OA versus NC, consisting of 12 up-regulated genes and 97 down-regulated genes (Figure 1B).

**Table 1:** The top 10 up- and down-regulated DEGs in RA and NC

Gene symbol	Fold-change	*P*-value
Top 10 up-regulated DEGs		
CXCL13	5.69921057	5.3239E-06
IGLC1	5.110816858	5.27729E-05
IGKV1-37	4.71661737	0.000103429
abParts	4.611513168	0.000251751
TNFRSF17	4.411048402	0.000180257
IGKV1OR2-108	4.206674027	0.000571657
CKAP2	4.197780218	0.000255427
GABBR1	4.107017141	2.79201E-06
PLA2G2D	3.900658676	0.000255118
IGKC	3.843207241	0.000189749
Top 10 down-regulated DEGs		
PCDHGA10	-4.684897922	1.96541E-06
NUMA1	-4.321247617	5.00011E-07
FKBP5	-3.694425306	2.39069E-05
ZBTB7C	-2.906578201	1.0086E-06
FBXW4P1	-2.692711395	5.55577E-08
SLC7A8	-2.613524388	1.88695E-06
COL6A1	-2.323616458	0.000212787
IL1RL2	-2.228749958	2.11044E-05
MIR612	-2.208297663	3.59695E-05
EGR1	-2.158797475	5.55606E-05

**Table 2:** The top 10 up- and down-regulated DEGs in OA and NC

Gene symbol	Fold-change	*P*-value
Top 10 up-regulated DEGs		
WIF1	3.311257345	0.002684048
SGCA	3.220848562	0.000699491
XIST	2.817303614	0.00213517
EPYC	2.506689631	6.51468E-05
C14orf105	2.458483277	0.00127672
WNT5B	2.361369339	0.001148016
NELL1	2.327586827	0.002937759
SCRG1	2.280146442	0.000171392
ZIC1	2.2690671	1.09591E-05
KCNK15	2.255434148	0.000113278
Top 10 down-regulated DEGs		
NUMA1	-4.485038149	1.4164E-06
PCDHGA10	-4.414593677	1.05336E-05
FKBP5	-3.368367204	2.33866E-05
MGC12488	-3.231687517	2.48311E-06
DDX3Y	-3.178836155	0.002532979
GUSBP3	-2.97556038	0.001170622
LINC00597	-2.965971219	0.000101076
KIAA0754	-2.965670459	0.00075988
MZT2B	-2.712432618	1.98934E-05
RUNX1-IT1	-2.707483939	0.000119435

**Table 3:** The top 10 up- and down-regulated DEGs in RA and OA

Gene symbol	Fold-change	*P*-value
Top 10 up-regulated DEGs		
HLA-DRB4	4.278036531	0.001561206
CXCL13	4.099735551	0.000509
PLA2G2D	3.941814384	0.000126896
CXCL9	3.432779798	3.16681E-07
GABBR1	3.15069778	2.72711E-06
ADAMDEC1	2.74998943	0.000109678
IL21R	2.715425603	5.72951E-05
PTPRCAP	2.648165742	0.001238936
CXCL10	2.384645081	2.90951E-05
SLAMF8	2.331058764	4.87428E-08
Top 10 down-regulated DEGs		
SGCA	-4.891082348	4.9407E-10
WIF1	-3.811093118	2.81802E-05
SCRG1	-3.248282523	1.13458E-07
RERGL	-2.956050594	0.001766259
STMN2	-2.931670338	3.23234E-05
ZIC1	-2.919357158	1.41272E-06
TOX3	-2.874222918	6.40796E-05
NELL1	-2.856181172	5.3584E-05
C7	-2.816039336	6.68233E-05
SGCG	-2.804064924	0.000109325

**Figure 2:**
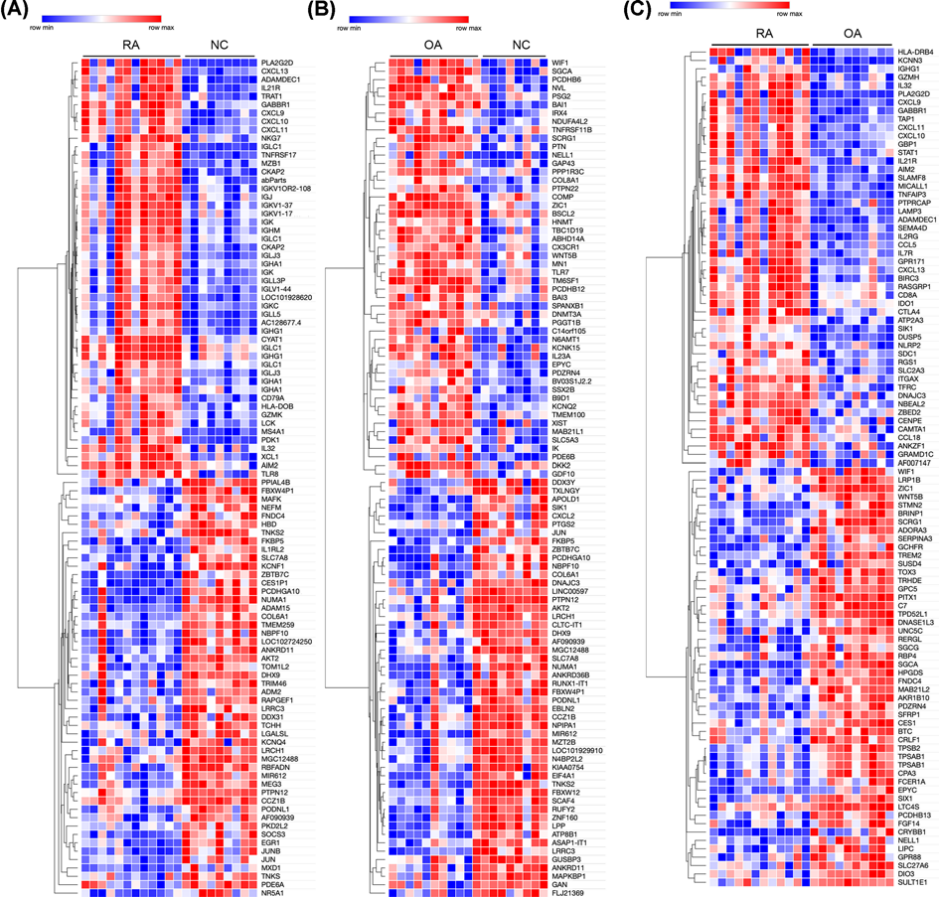
Clustering heatmap of the genes exhibiting significantly differential expression (**A**) RA versus NC group, (**B**) OA versus NC group and (**C**) RA versus OA group. Statistically significant DEGs were defined as |log2Foldchange| > 1 and *P*-value < 0.05. DEG, differentially expressed gene; OA, osteoarthritis; RA, rheumatoid arthritis; NC, normal controls.

The volcano plot of DEGs in each group was presented in Figure 1C–E. In addition, Using the Morpheus website, we developed a clustering heatmap of the DEGs (Figure 2A,B).

We verified the top 10 up- and down-regulated genes in GSE55235. As shown in Supplementary Table S1, except for SGCG, all the other genes are differentially expressed between synovial tissue from RA and OA. The Venn diagrams also showed the 24 overlap DEGs between synovial tissue and peripheral blood from RA and OA, the 10 overlap DEGs between synovial tissue and bone marrow from RA and OA in GSE12021 and GSE100786 (Supplementary Figure S1).

### Enrichment analysis of DEGs

**Figure 3:**
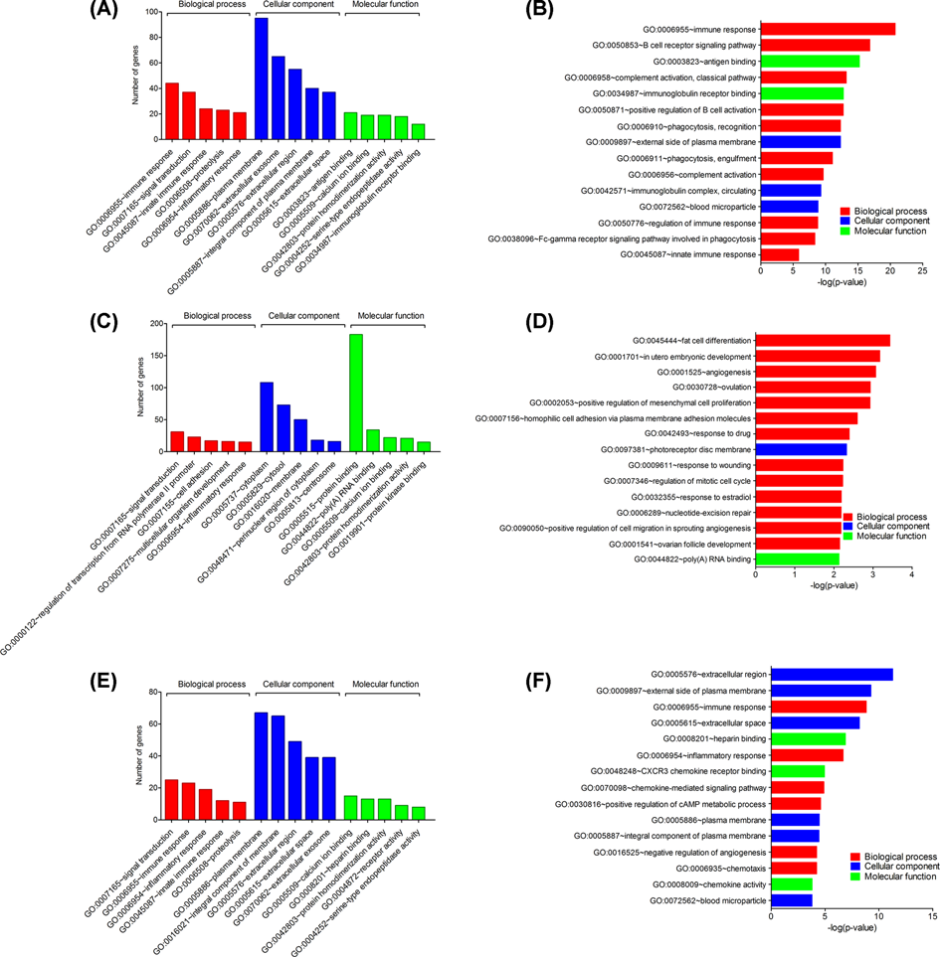
Gene ontology (GO) enrichment of the DEGs The DEGs in RA versus NC group sorted by descending order of the number of genes (**A**) and −Log10(*P*-value) (**B**). The DEGs in OA versus NC group sorted by descending order of the number of genes (**C**) and −Log10(*P*-value) (**D**). The DEGs in RA versus OA group sorted by descending order of the number of genes (**E**) and −Log10(*P*-value) (**F**); NC, normal controls; OA, osteoarthritis; RA, rheumatoid arthritis.

**Figure 4:**
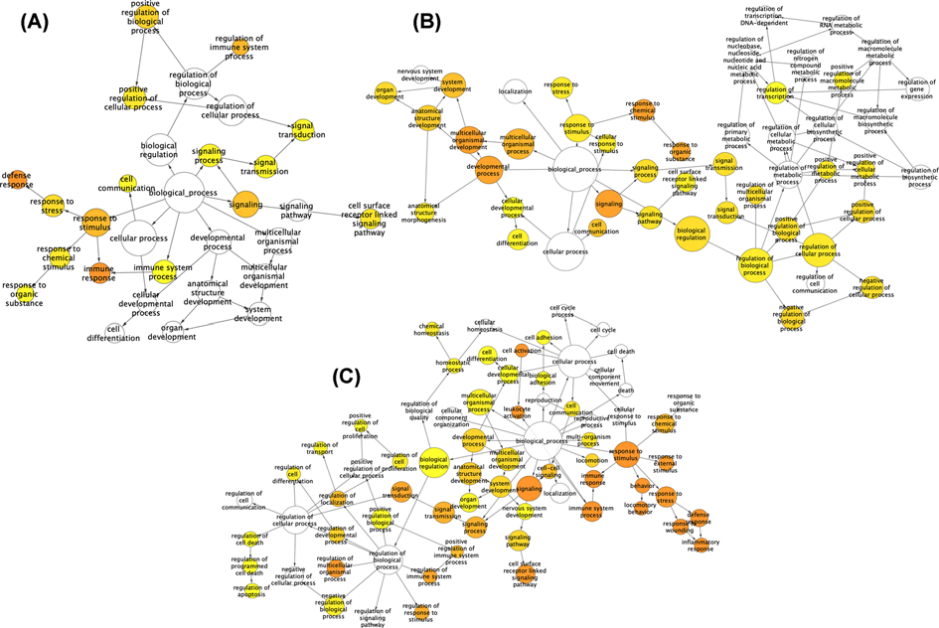
The biological process of the DEGs analyzed by BiNGO (**A**) RA versus NC group, (**B**) OA versus NC group and (**C**) RA versus OA group. The color depth of nodes represents the corrected *P*‐value. The size of nodes represents the number of genes involved; DEG, differentially expressed gene; NC, normal controls; OA, osteoarthritis; RA, rheumatoid arthritis.

**Figure 5:**
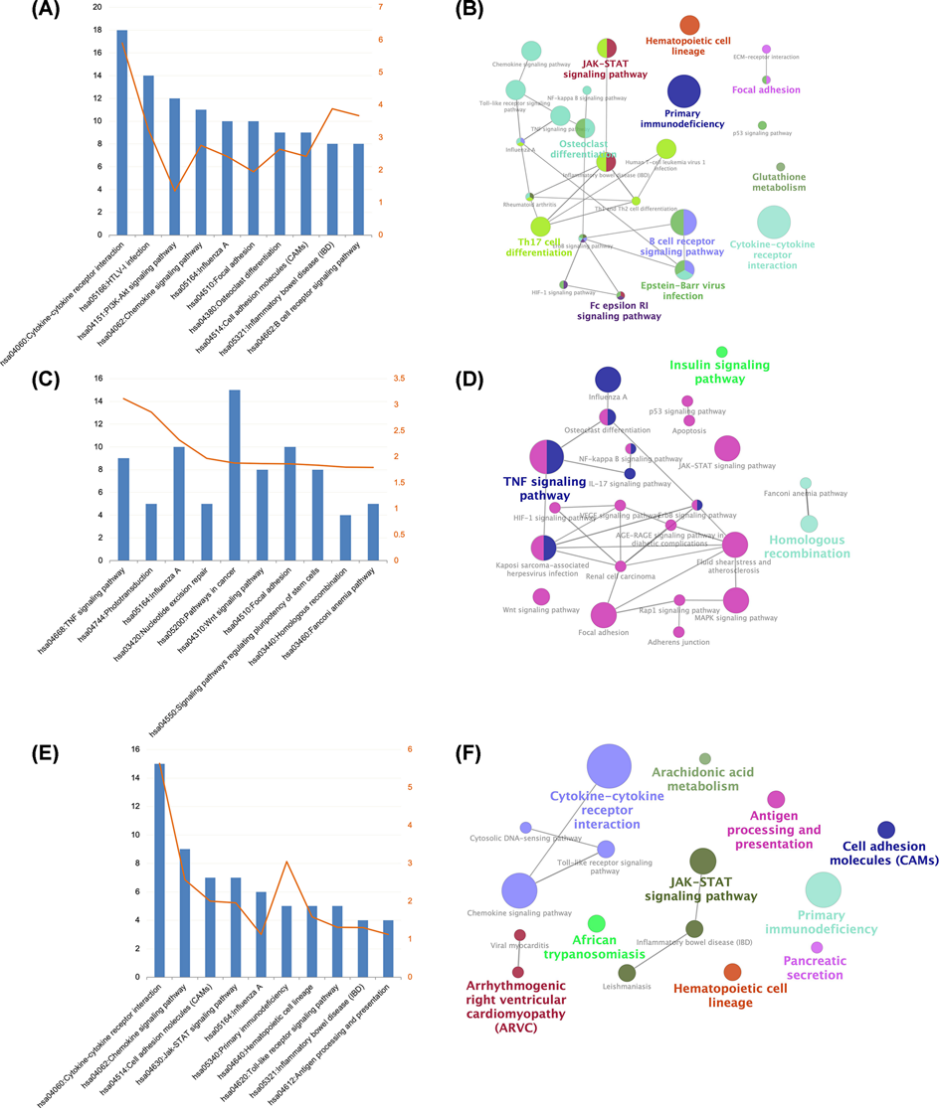
KEGG pathways enriched by the DEGs and interrelation analysis between pathways molecular functions (**A,B**) RA versus NC group, (**C,D**) OA versus NC group and (**E,F**) RA versus OA group; DEG, differentially expressed gene; KEGG, Kyoto Encyclopedia of Genes and Genomes; NC, normal controls; OA, osteoarthritis; RA, rheumatoid arthritis.

To gain insights into the biological roles of the DEGs, we performed GO categories enrichment analysis. GO term enrichment analysis results varied according to GO classification and expression change of DEGs. With the criterion of *P*<0.05, ‘immune response’ (RA vs. NC group), ‘signal transduction’, ‘fat cell differentiation’ (OA vs. NC group), and ‘signal transduction’, ‘immune response’ (RA vs. OA group) exhibited highly significant enrichment within the GO biological process category. For the cellular component category, DEGs were significantly enriched in ‘plasma membrane’, ‘external side of plasma membrane’ (RA vs. NC group), ‘cytoplasm’, ‘photoreceptor disc membrane’ (OA vs. NC group), and ‘plasma membrane’, ‘extracellular region’ (RA vs. OA group). In addition, the molecular function category contained DEGs significantly enriched in ‘antigen binding’ (RA vs. NC group), ‘protein binding’, ‘poly(A) binding’ (OA vs. NC group), and ‘calcium ion binding’, ‘heparin binding’ (RA vs. OA group) (Figures 3 and 4; Supplementary Figures S2–4). The top enriched KEGG pathways included ‘cytokine–cytokine receptor interaction’ (RA vs. NC group), ‘TNF signaling pathway’ (OA vs. NC group), and ‘cytokine–cytokine receptor interaction’ (RA vs. OA group) (Figure 5, Supplementary Tables S2–4).

**Figure 6:**
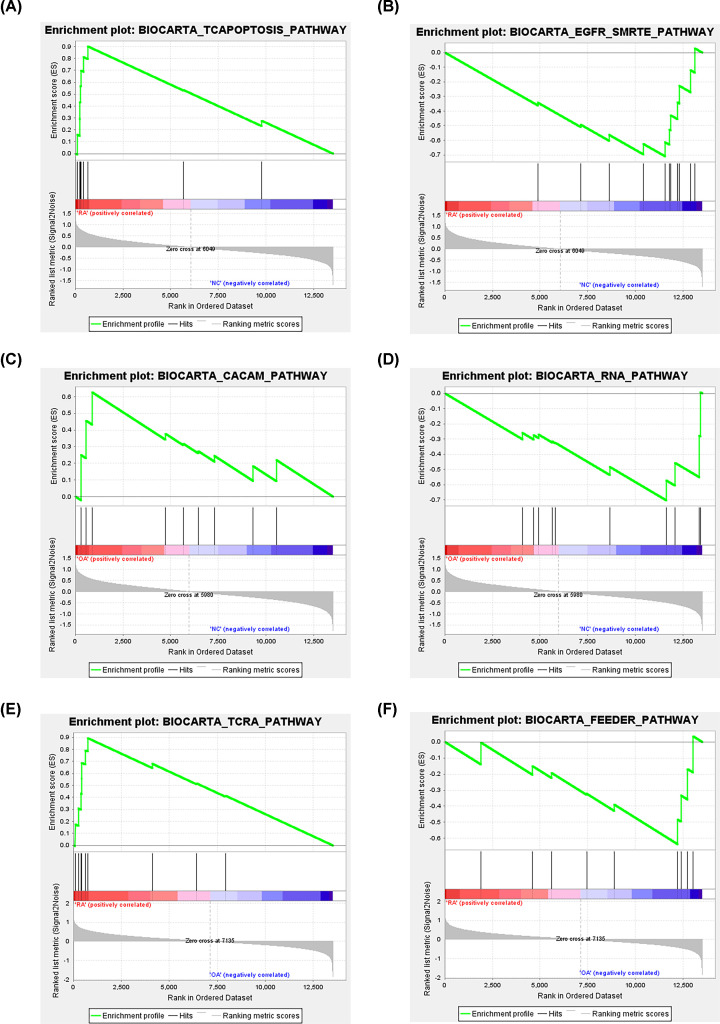
Gene set enrichment analysis (GSEA) (**A,B**) RA versus NC group, (**C,D**) OA versus NC group and (**E,F**) RA versus OA group.

Additionally, we used the DEGs to perform GSEA analysis (Figure 6). TCRA pathway, FEEDER pathway (RA vs. OA group), EGFR_SMRTE pathway, TCAPOPTOSIS pathway (RA vs. NC group), CACAM pathway, RNA pathway (OA vs. NC group) were significantly enriched (Figure 6).

### PPI network analysis of DEGs

To further explore the relationships between DEGs at the protein level, the PPI networks were constructed based on the interactions of DEGs. In total, 154 nodes and 318 interactions (RA vs. NC group), 136 nodes and 118 interactions (OA vs. NC group), and 177 nodes and 275 interactions (RA vs. OA group) were screened to establish the PPI network. In this network, the top 10 key genes with highest degree scores are shown in Table 4.

**Table 4:** The top 15 hub genes with highest degree scores

RA versus NC	Degree	OA versus NC	Degree	RA versus OA	Degree
CD19	28	JUN	15	PTPRC	27
LCK	22	CTNNB1	15	ITGAX	24
CD2	22	DICER1	8	CTLA4	23
CD27	22	SIL1	7	CCL5	21
CXCL10	19	TLR7	7	CXCL10	20
CCL5	19	DHX9	7	STAT1	20
CD79A	18	RPL27A	7	CXCR3	18
CD79B	18	SOCS3	6	APOE	17
CXCL9	17	RPL35A	6	CXCL9	16
IL2RG	17	INSR	6	IL7R	15

### Module analysis

**Figure 7:**
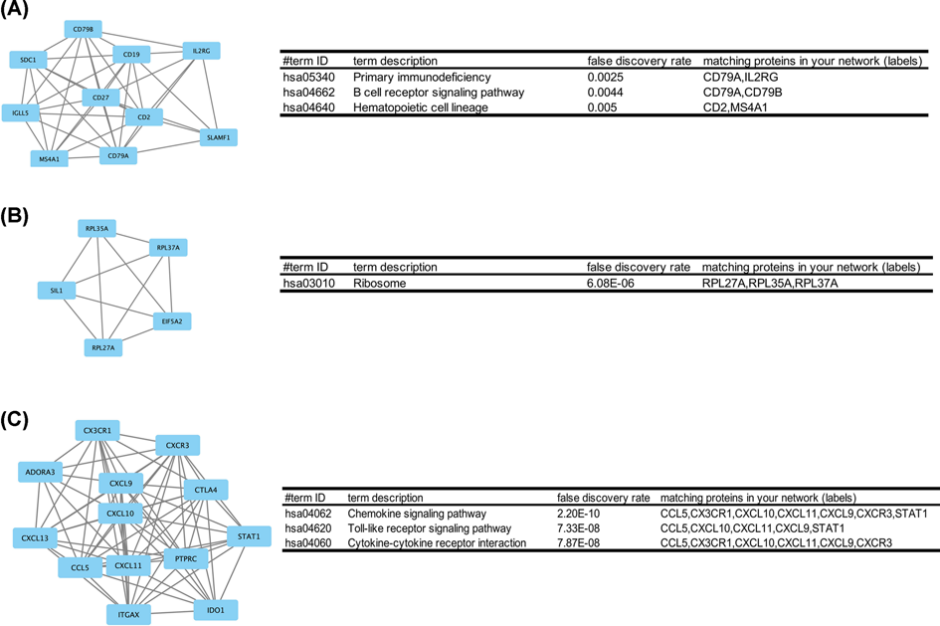
Top modules from the protein-protein interaction network RA versus NC group: (**A**) module, (**B**) the KEGG enriched pathways of module, OA versus NC group: (**C**) module, (**D**) the KEGG enriched pathways of module, RA versus OA group: (**E**) module, (**F**) the KEGG enriched pathways of module; KEGG, Kyoto Encyclopedia of Genes and Genomes; NC, normal controls; OA, osteoarthritis; RA, rheumatoid arthritis.

The top significant modules were selected respectively, and functional annotation of the genes from the modules was analyzed (Figure 7). KEGG enrichment analysis showed that the genes were mainly associated with ‘primary immunodeficiency’ (RA vs. NC group), ‘ribosome’ (OA vs. NC group), and ‘chemokine signaling pathway’ (RA vs. OA group) (Figure 7).

### Identification of related active small molecules

DEGs were first divided into up-regulated and down-regulated groups and then enriched with significantly changed genes obtained from treatment of small molecules from the CMap database. Table 5 showed the predicted small molecules that could inhibit RA/OA-associated gene expression. Cefamandole and Arecoline were identified in OA and RA tissue analysis.

**Table 5:** List of the 10 most significant small molecule drugs that can reverse the tumoral status of RA and OA

Arthritic tissue	CMap name	enrichment	p value
RA versus NC	Cefamandole	-0.939	0
	Arachidonyltrifluoromethane	-0.869	0.03438
	Quipazine	-0.85	0.00095
	Isometheptene	-0.847	0.00101
	Nadolol	-0.825	0.00177
	3-Acetamidocoumarin	-0.824	0.00185
	Prestwick-1082	-0.815	0.0125
	Aciclovir	-0.809	0.00014
	Canadine	-0.809	0.00259
	Gentamicin	-0.783	0.00444
OA versus NC	Arecoline	-0.889	0.00034
	Sulmazole	-0.885	0.00298
	Prestwick-983	-0.875	0.00393
	Ciclopirox	-0.853	0.00084
	Prestwick-691	-0.835	0.00891
	Prestwick-692	-0.829	0.00163
	SR-95639A	-0.823	0.00189
	Lasalocid	-0.81	0.00257
	Clemastine	-0.804	0.01498
	Meclocycline	-0.793	0.00372

## Discussion

OA and RA are two most common joint diseases with similar characteristics in synovitis. However, the underlying pathogenesis of RA and OA remain unclear. Clarifying the molecular mechanism differences between the above two diseases will help to define special targets for therapeutic intervention. In the present study, bioinformatics analysis was performed to analyze the underlying molecular mechanisms differences between the above two diseases.

We extracted the data from GSE12021, which including 9 NC, 12 RA, and 10 OA samples. We identified 165 up-regulated and 163 down-regulated DEGs between RA and NC samples, 73 up-regulated and 293 down-regulated DEGs between OA and NC samples, 92 up-regulated and 98 down-regulated DEGs between RA and OA samples using bioinformatics analysis. We also reanalyzed the expression of the DEGs in GSE55235 and verified the DEGs in human PB and 8 BM monocytes samples from dataset GSE100786. The GO and KEGG functional annotation and pathway enrichment analyses suggested that the identified DEGs were mainly involved in ‘immune response’, ‘cytokine–cytokine receptor interaction’ (RA vs. NC group), ‘signal transduction’, ‘fat cell differentiation’ and ‘TNF signaling pathway’ (OA vs. NC group), and ‘signal transduction’, ‘immune response’, and ‘cytokine–cytokine receptor interaction’ (RA vs. OA group).

RA is an autoimmune disease featured with pain, swelling, and destruction of synovial joints, leading to functional disability [11]. Inflammatory and immune response result in excessive secretion of inflammatory cytokines, growth factors, and matrix metalloproteinases (MMPs), resulting in synovitis and joints degradation [12]. Chemokines and chemokine receptors participate in cellular migration, survival, angiogenesis and leukocyte recruitment of RA and other autoimmune diseases [13]. Based on this present study, CD19, CD2, CD27, CD79A, CD79B, CXCL10, and CXCL9 were immune‐related RA‐specific DEGs, which might play important roles in the pathogenesis of RA. Although the pathogenesis of RA is not fully understood, leukocyte migration, which is regulated part by cytokines and cytokine receptors contribute to the perpetuation of synovium inflammation in RA. Cytokine–cytokine receptor interaction was a significantly enriched pathway for RA‐specific DEGs that emphasized the importance of Cytokine-cytokine receptor interaction and its related RA‐specific DEGs in the pathogenesis of RA.

Tumor necrosis factor α (TNF‐α) is a potent proinflammatory cytokine that plays a crucial role in inflammatory and immune responses as well as in the pathogenesis of OA. In addition, TNF-α can enhance cartilage degradation and induce bone resorption in OA [14–16]. Based on this present study, TLR7 and SOCS3 were two TNF‐related OA‐specific DEGs which might play roles in the pathogenesis of OA. The activation of the TLR7/8 signaling pathway led to the activation of NF-κB which was required for the induction of TNF-α [17]. SOCS3, as a suppressor of cytokine signaling, has been implicated in transcriptional activation of signal transduction and activators of transcription (STAT) signaling pathway [18–20]. SOCS3 is up-regulated in response to various cytokines such as TNF-α, IL-6 and growth hormone [21].

The secretion of CXCL9/10 by immune cells is dependent on IFN-γ [22]. The high level of CXCL9/10 in peripheral liquids is a biomarker of immune response, especially involving Th1-cells. Moreover, the CXCL10 concentrations in RA synovial fluid are much higher than those in OA [23]. Many previous studies revealed the significant role of CXCL9 and CXCL10 in the inflammatory process in RA. In this present study, CXCL9 and CXCL10 were found to be up-regulated in RA compared with both OA and normal controls which provided evidence for the previous study and suggested that they might be potential biomarkers for discrimination of RA and OA.

More importantly, based on the DEGs and data from the CMap database, we acquired a series of small molecules. We were surprised to find that among these small molecules, Cefamandole and Arecoline were demonstrated significant similarity in RA and OA tissues (*P*<0.05) and additional analysis was required to determine their suitability as broad spectrum anti-arthritis drugs.

Arecoline (methyl-1, 2, 5, 6-tetrahydro-1-methyl-nicotinate) is an alkaloid isolated from *Areca catechu*, and it is considered as the major effective constituent of *A. catechu* [24, 25]. Recently research investigated the potential pharmacological and toxic effects of arecoline [26, 27]. The main toxic effects of arecoline are oral submucous fibrosis (OSF), oral squamous cell carcinoma (OSCC) and genotoxicity [28–30]. Cefamandole has been recommended in empiric therapy for patients with community-acquired pneumonia and as a prophylactic agent for patients receiving various surgical procedures [31].

In summary, we provide bioinformatic evidence demonstrating that CXCL9 and CXCL10 might be potential biomarkers for discrimination of RA and OA. Additionally, Cefamandole and Arecoline were demonstrated as the potential anti-arthritis drugs in RA and OA tissues (*P*<0.05). As the pathogenic mechanisms of RA and OA are still not clear, our discoveries may have a broad impact in RA and OA biology and therapy. Nevertheless, large sample size and further mechanism experiments are still needed to confirm our conclusion.

## Supplementary Material

Supplementary Figure S1 and Tables S1-S4

## Abbreviations

BM: Bone marrow
CMap: the Connectivity Map
DEG: Differentially expressed gene
FC: Fold change
GEO: Gene Expression Omnibus
GO: Gene ontology
GSEA: Gene set enrichment analysis
MCODE: The Molecular Complex Detection
NC: Normal control
OA: Osteoarthritis
OSCC: Oral squamous cell carcinoma
PB: Peripheral blood
PPI: Protein–protein interaction
RA: Rheumatoid arthritis
STAT: Signal transduction and activators of transcription
TNF‐α: Tumor necrosis factor α

## References

[1] Hermann W., Lambova S. and Muller-Ladner U. (2018) Current Treatment Options for Osteoarthritis. Curr. Rheumatol. Rev. 14, 108–116 10.2174/1573397113666170829155149 28875826

[2] Kohler B.M., Gunther J., Kaudewitz D. and Lorenz H.M. (2019) Current Therapeutic Options in the Treatment of Rheumatoid Arthritis. J. Clin. Med. 8, 938 10.3390/jcm8070938 PMC6678427 31261785

[3] Baecklund E., Iliadou A., Askling J. et al. (2006) Association of chronic inflammation, not its treatment, with increased lymphoma risk in rheumatoid arthritis. Arthritis Rheum. 54, 692–701 10.1002/art.21675 16508929

[4] Franklin J., Lunt M., Bunn D. et al. (2006) Incidence of lymphoma in a large primary care derived cohort of cases of inflammatory polyarthritis. Ann. Rheum. Dis. 65, 617–622 10.1136/ard.2005.044784 16249224 PMC1798140

[5] Turkiewicz A., Neogi T., Bjork J. et al. (2016) All-cause Mortality in Knee and Hip Osteoarthritis and Rheumatoid Arthritis. Epidemiology 27, 479–485 10.1097/EDE.0000000000000477 26986874

[6] Ernst E. and Posadzki P. (2011) Complementary and alternative medicine for rheumatoid arthritis and osteoarthritis: an overview of systematic reviews. Curr. Pain Headache Rep. 15, 431–437 10.1007/s11916-011-0227-x 21979101

[7] Li Z.C., Xiao J., Peng J.L. et al. (2014) Functional annotation of rheumatoid arthritis and osteoarthritis associated genes by integrative genome-wide gene expression profiling analysis. PLoS ONE 9, e85784 10.1371/journal.pone.0085784 24551036 PMC3925090

[8] Zhu N., Hou J., Wu Y. et al. (2018) Identification of key genes in rheumatoid arthritis and osteoarthritis based on bioinformatics analysis. Medicine (Baltimore). 97, e10997 10.1097/MD.0000000000010997 29851858 PMC6392928

[9] Huber R., Hummert C., Gausmann U. et al. (2008) Identification of intra-group, inter-individual, and gene-specific variances in mRNA expression profiles in the rheumatoid arthritis synovial membrane. Arthritis Res. Ther. 10, R98 10.1186/ar2485 18721452 PMC2575612

[10] Cai P., Jiang T., Li B. et al. (2019) Comparison of rheumatoid arthritis (RA) and osteoarthritis (OA) based on microarray profiles of human joint fibroblast-like synoviocytes. Cell Biochem. Funct. 37, 31–41 10.1002/cbf.3370 30468518

[11] Toquet S., Nguyen Y., Sabbagh A. et al. (2016) Severe apoptotic enteropathy caused by methotrexate treatment for rheumatoid arthritis. Joint Bone Spine 83, 217–219 10.1016/j.jbspin.2015.08.006 26494588

[12] Wei Y., Sun X., Hua M. et al. (2015) Inhibitory Effect of a Novel Antirheumatic Drug T-614 on the IL-6-Induced RANKL/OPG, IL-17, and MMP-3 Expression in Synovial Fibroblasts from Rheumatoid Arthritis Patients. Biomed. Res. Int. 2015, 214683 10.1155/2015/214683 26273599 PMC4530218

[13] Szekanecz Z. and Koch A.E. (2016) Successes and failures of chemokine-pathway targeting in rheumatoid arthritis. Nat. Rev. Rheumatol. 12, 5–13 10.1038/nrrheum.2015.157 26607389

[14] Pelletier J.P., Martel-Pelletier J. and Abramson S.B. (2001) Osteoarthritis, an inflammatory disease: potential implication for the selection of new therapeutic targets. Arthritis Rheum. 44, 1237–1247 10.1002/1529-0131(200106)44:6<1237::AID-ART214>3.0.CO;2-F 11407681

[15] Huebner J.L., Seifer D.R. and Kraus V.B. (2007) A longitudinal analysis of serum cytokines in the Hartley guinea pig model of osteoarthritis. Osteoarthritis Cartilage 15, 354–356 10.1016/j.joca.2006.10.014 17208467 PMC1852468

[16] Kobayashi M., Squires G.R., Mousa A. et al. (2005) Role of interleukin-1 and tumor necrosis factor alpha in matrix degradation of human osteoarthritic cartilage. Arthritis Rheum. 52, 128–135 10.1002/art.20776 15641080

[17] Lee J., Tian Y., Chan S.T. et al. (2015) TNF-alpha Induced by Hepatitis C Virus via TLR7 and TLR8 in Hepatocytes Supports Interferon Signaling via an Autocrine Mechanism. PLoS Pathog. 11, e1004937 10.1371/journal.ppat.1004937 26023919 PMC4449221

[18] Mesaki K., Yamane M., Sugimoto S. et al. (2019) SOCS3 overexpression in T cells ameliorates chronic airway obstruction in a murine heterotopic tracheal transplantation model. Surg. Today 49, 443–450 10.1007/s00595-018-1753-5 30617600

[19] Yong Y.H., Wang P., Jia R.M. et al. (2019) SOCS3 control the activity of NF-kappaB induced by HSP70 via degradation of MyD88-adapter-like protein (Mal) in IPEC-J2 cells. Int. J. Hyperthermia 36, 151–159 10.1080/02656736.2018.1541484 30484725

[20] Pedroso J.A.B., Ramos-Lobo A.M. and Donato J. Jr (2019) SOCS3 as a future target to treat metabolic disorders. Hormones (Athens) 18, 127–136 10.1007/s42000-018-0078-5 30414080

[21] Zhao X., Qi R., Sun C. and Xie Y. (2012) Silencing SOCS3 could inhibit TNF-alpha induced apoptosis in 3T3-L1 and mouse preadipocytes. Mol. Biol. Rep. 39, 8853–8860 10.1007/s11033-012-1749-y 22714916

[22] Jinquan T., Jing C., Jacobi H.H. et al. (2000) CXCR3 expression and activation of eosinophils: role of IFN-gamma-inducible protein-10 and monokine induced by IFN-gamma. J. Immunol. 165, 1548–1556 10.4049/jimmunol.165.3.1548 10903763

[23] Lee E.Y., Seo M., Juhnn Y.S. et al. (2011) Potential role and mechanism of IFN-gamma inducible protein-10 on receptor activator of nuclear factor kappa-B ligand (RANKL) expression in rheumatoid arthritis. Arthritis Res. Ther. 13, R104 10.1186/ar3385 21708014 PMC3218919

[24] Shih Y.T., Chen P.S., Wu C.H. et al. (2010) Arecoline, a major alkaloid of the areca nut, causes neurotoxicity through enhancement of oxidative stress and suppression of the antioxidant protective system. Free Radic. Biol. Med. 49, 1471–1479 10.1016/j.freeradbiomed.2010.07.017 20691257

[25] Cai Z.Y., Li Y.C., Li L.H. and Chen Z.G. (2012) Analysis of arecoline in Semen Arecae decoction pieces by microchip capillary electrophoresis with contactless conductivity detection. J. Pharm. Anal. 2, 356–360 10.1016/j.jpha.2012.07.003 29403766 PMC5760757

[26] Saha I., Chakraborty S.B., Chatterjee A. et al. (2018) Arecoline inhibits pineal-testis function in experimentally induced hypothyroid rats. Arch. Physiol. Biochem. 126, 1–10 30145920 10.1080/13813455.2018.1486428

[27] Chang C.H., Chen M.C., Chiu T.H. et al. (2019) Arecoline Promotes Migration of A549 Lung Cancer Cells through Activating the EGFR/Src/FAK Pathway. Toxins (Basel) 11, 185 10.3390/toxins11040185 PMC6521018 30925742

[28] Yao M., Li J., Yuan S. et al. (2019) Role of the arecoline/YAP1/BMP4 pathway in promoting endothelial-mesenchymal transition in oral submucous fibrosis. J. Oral. Pathol. Med. 49, 305–310 31397922 10.1111/jop.12945

[29] Dai Z., Zhu B., Yu H. et al. (2019) Role of autophagy induced by arecoline in angiogenesis of oral submucous fibrosis. Arch. Oral. Biol. 102, 7–15 10.1016/j.archoralbio.2019.03.021 30951892

[30] Kuo T.M., Nithiyanantham S., Lee C.P. et al. (2019) Arecoline N-oxide regulates oral squamous cell carcinoma development through NOTCH1 and FAT1 expressions. J. Cell. Physiol. 234, 13984–13993 10.1002/jcp.28084 30624777 PMC13483058

[31] Sanders C.V., Greenberg R.N. and Marier R.L. (1985) Cefamandole and cefoxitin. Ann. Intern. Med. 103, 70–78 10.7326/0003-4819-103-1-70 3890658

