# Supplementary material for: Identification of novel biomarkers and candidate small molecule drugs in rheumatoid arthritis and osteoarthritis based on bioinformatics analysis of high‐throughput data
Source: Biosci Rep. 2020 Dec 16;40(12):BSR20193823. doi: 10.1042/BSR20193823 (PMC7744737; doi:10.1042/BSR20193823)
Supplement: Supplementary Figure S1 and Tables S1-S4 [file BSR-2019-3823_supp.pdf]

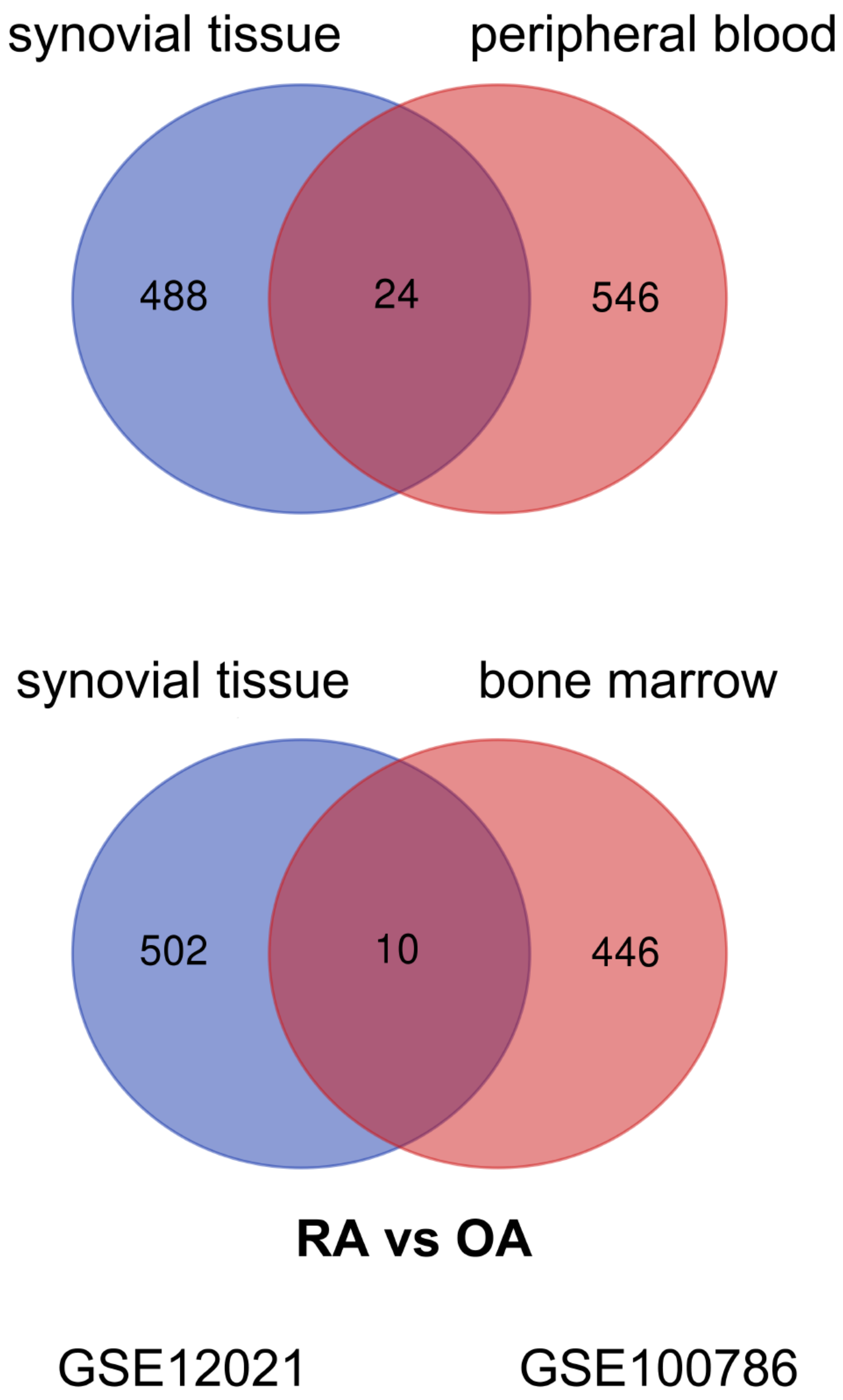
synovial tissue
peripheral blood
488
24
546
synovial tissue
bone marrow
502
10
446
RA vs OA
GSE12021
GSE100786

Supplementary Table 1 The top 10 DEGs in RA and NC were verified in GSE55235.

Gene symbol	Fold-change	P-value
HLA-DRB4	2.745061813	0.020334961
CXCL13	6.184201362	1.33441E-09
PLA2G2D	2.648396684	0.000363523
CXCL9	2.564950266	5.27067E-06
GABBR1	3.965429039	1.26869E-07
ADAMDEC1	3.294369641	1.11079E-09
IL21R	1.837310467	1.96539E-06
PTPRCAP	2.100081267	7.72729E-06
CXCL10	1.976383233	6.13273E-06
SLAMF8	2.219171944	1.60026E-09
SGCA	-1.627227378	0.000103414
WIF1	-2.626945213	0.000327803
SCRG1	-2.41571327	5.82003E-06
RERGL	-2.020052254	6.4712E-05
STMN2	-1.692069842	0.000842938
ZIC1	-3.779380032	0.00017211
TOX3	-2.908460707	0.002935494
NELL1	-1.762191949	0.001163006
C7	-2.853407158	8.59428E-06
SGCG	-0.305196375	0.595344054

upplementary Table 2 Functional and pathway enrichment analysis of the DEGs in RA vs NC group.

Category	Term	Count	PValue
GOTERM_BP_DIRECT	GO:0006955~immune response	44	1.80E-21
GOTERM_BP_DIRECT	GO:0050853~B cell receptor signaling pathway	18	1.46E-17
GOTERM_BP_DIRECT	GO:0006958~complement activation, classical pathway	19	6.59E-14
GOTERM_BP_DIRECT	GO:0050871~positive regulation of B cell activation	12	1.85E-13
GOTERM_BP_DIRECT	GO:0006910~phagocytosis, recognition	12	4.98E-13
GOTERM_CC_DIRECT	GO:0009897~external side of plasma membrane	25	5.00E-13
GOTERM_CC_DIRECT	GO:0042571~immunoglobulin complex, circulating	9	5.29E-10
GOTERM_CC_DIRECT	GO:0072562~blood microparticle	18	1.51E-09
GOTERM_CC_DIRECT	GO:0005576~extracellular region	55	2.71E-06
GOTERM_CC_DIRECT	GO:0005886~plasma membrane	95	0.00274657
GOTERM_MF_DIRECT	GO:0003823~antigen binding	21	6.11E-16
GOTERM_MF_DIRECT	GO:0034987~immunoglobulin receptor binding	12	1.81E-13
GOTERM_MF_DIRECT	GO:0008009~chemokine activity	9	1.60E-06
GOTERM_MF_DIRECT	GO:0004252~serine-type endopeptidase activity	18	1.90E-06
GOTERM_MF_DIRECT	GO:0048248~CXCR3 chemokine receptor binding	4	4.79E-05
KEGG_PATHWAY	hsa04060:Cytokine-cytokine receptor interaction	18	1.24E-06
KEGG_PATHWAY	hsa05340:Primary immunodeficiency	7	2.58E-05
KEGG_PATHWAY	hsa05321:Inflammatory bowel disease (IBD)	8	1.31E-04
KEGG_PATHWAY	hsa04662:B cell receptor signaling pathway	8	2.12E-04
KEGG_PATHWAY	hsa05166:HTLV-I infection	14	5.71E-04

upplementary Table 3 Functional and pathway enrichment analysis of the DEGs in OA vs NC group.

Category	Term	Count	PValue
GOTERM_BP_DIRECT	GO:0045444~fat cell differentiation	8	3.70E-04
GOTERM_BP_DIRECT	GO:0001701~in utero embryonic development	12	6.71E-04
GOTERM_BP_DIRECT	GO:0001525~angiogenesis	13	8.53E-04
GOTERM_BP_DIRECT	GO:0030728~ovulation	4	0.00117787
GOTERM_BP_DIRECT	GO:0002053~positive regulation of mesenchymal cell proliferation	5	0.00119207
GOTERM_CC_DIRECT	GO:0097381~photoreceptor disc membrane	4	0.00475457
GOTERM_CC_DIRECT	GO:0000439~core TFIIH complex	3	0.01107251
GOTERM_CC_DIRECT	GO:0042995~cell projection	6	0.01217713
GOTERM_CC_DIRECT	GO:0005813~centrosome	16	0.01248841
GOTERM_CC_DIRECT	GO:0005923~bicellular tight junction	7	0.01784125
GOTERM_MF_DIRECT	GO:0044822~poly(A) RNA binding	34	0.00738839
GOTERM_MF_DIRECT	GO:0019901~protein kinase binding	15	0.01165975
GOTERM_MF_DIRECT	GO:0005515~protein binding	183	0.02608831
GOTERM_MF_DIRECT	GO:0005509~calcium ion binding	22	0.02908331
GOTERM_MF_DIRECT	GO:0005251~delayed rectifier potassium channel activity	4	0.02942848
KEGG_PATHWAY	hsa04668:TNF signaling pathway	9	7.64E-04
KEGG_PATHWAY	hsa04744:Phototransduction	5	0.00139913
KEGG_PATHWAY	hsa05164:Influenza A	10	0.00473497
KEGG_PATHWAY	hsa03420:Nucleotide excision repair	5	0.01071053
KEGG_PATHWAY	hsa05200:Pathways in cancer	15	0.01321574

upplementary Table 4 Functional and pathway enrichment analysis of the DEGs in RA vs OA group.

Category	Term	Count	PValue
GOTERM_BP_DIRECT	GO:0006955~immune response	23	1.50E-09
GOTERM_BP_DIRECT	GO:0006954~inflammatory response	19	2.12E-07
GOTERM_BP_DIRECT	GO:0070098~chemokine-mediated signaling pathway	8	1.29E-05
GOTERM_BP_DIRECT	GO:0030816~positive regulation of cAMP metabolic process	4	2.57E-05
GOTERM_BP_DIRECT	GO:0016525~negative regulation of angiogenesis	7	6.07E-05
GOTERM_CC_DIRECT	GO:0005576~extracellular region	49	5.28E-12
GOTERM_CC_DIRECT	GO:0009897~external side of plasma membrane	17	5.58E-10
GOTERM_CC_DIRECT	GO:0005615~extracellular space	39	6.60E-09
GOTERM_CC_DIRECT	GO:0005886~plasma membrane	67	3.46E-05
GOTERM_CC_DIRECT	GO:0005887~integral component of plasma membrane	32	3.73E-05
GOTERM_MF_DIRECT	GO:0008201~heparin binding	13	1.29E-07
GOTERM_MF_DIRECT	GO:0048248~CXCR3 chemokine receptor binding	4	1.14E-05
GOTERM_MF_DIRECT	GO:0008009~chemokine activity	6	1.61E-04
GOTERM_MF_DIRECT	GO:0004896~cytokine receptor activity	5	5.41E-04
GOTERM_MF_DIRECT	GO:0004872~receptor activity	9	0.00216207
KEGG_PATHWAY	hsa04060:Cytokine-cytokine receptor interaction	15	2.33E-06
KEGG_PATHWAY	hsa05340:Primary immunodeficiency	5	9.03E-04
KEGG_PATHWAY	hsa04062:Chemokine signaling pathway	9	0.00267603
KEGG_PATHWAY	hsa04514:Cell adhesion molecules (CAMs)	7	0.00994524
KEGG_PATHWAY	hsa04630:Jak-STAT signaling pathway	7	0.01095865
